# Peek a boo! Information seeking about food and functionality in capuchin monkeys

**DOI:** 10.1007/s10071-025-01999-2

**Published:** 2025-10-30

**Authors:** E. J. Jordan, M. Allritz, M. Bohn, C. J. Völter, Amanda M. Seed

**Affiliations:** 1 School of Psychology and Neuroscience, University of St Andrews, Scotland, UK; 2 Department of Comparative Cultural Psychology, Max Planck Institute for Evolutionary Anthropology, Deutscher Platz 6, Leipzig, Germany; 3 Institute of Psychology in Education, Leuphana University, Lüneburg Universitätsallee 1, 21335 Lüneburg, Germany; 4 Comparative Cognition, Messerli Research Institute, University of Veterinary Medicine Vienna, Medical University of Vienna and University of Vienna, Vienna, Austria

**Keywords:** Capuchin monkeys, Primates, Metacognition, Information-seeking, Strategic search

## Abstract

The ability to be aware of your own knowledge state (metacognition) can be investigated by examining an individual’s information-seeking behaviour. Chimpanzees (Pan troglodytes) perform strategic searches for food and tools. However, although capuchin monkeys (Sapajus apella) seek information about food, whether they search for functional information is unknown. Further, if information seeking indicates awareness of what knowledge is missing, rather than an uncertainty response, search patterns should reflect the missing information. We presented 12 capuchin monkeys with two novel information seeking tasks; Experiments 1 and 2 investigated their food search, Experiment 3 investigated their search for functionality. In both tasks information could be sought from two locations; looking below a barrier provided information about food, looking above a barrier provided information about food in Experiments 1 and 2 or cup functionality (open or sealed) in Experiment 3. Monkeys were trained to select the target cup with all information visible. Then we occluded the cups and presented the monkeys with different configurations of missing information requiring looks above, below, or both to locate the target cup. When searching for both food and functional information the monkeys’ searching was selective; it was more likely under occluded conditions. However, search location was not significantly affected by different configurations of missing information, suggesting they were not tailoring their information seeking. This supports previous findings that capuchins perform information-seeking to fill knowledge gaps. However, we found no evidence that searching was sensitive to the information was required. We conclude that capuchin monkeys show selective but not strategic information seeking.

**Supplementary Information:**

The online version contains supplementary material available at 10.1007/s10071-025-01999-2.

## Introduction

Whether for food, mates, or shelter (to name just a few possibilities), many animals engage in information seeking. It has been proposed that in some situations this information seeking could serve as an indicator of metacognition (Call and Carpenter 2001). For example, if an individual is selective in its information seeking (i.e. only searching for information when it is missing) it suggests that the individual is sensitive to the fact that they lack information. However, other researchers argue that selective information seeking in non-humans can be explained by a simple response to uncertainty (Carruthers and Williams 2019; Crystal and Foote 2011). Although this may be true in some cases, search routines may also be triggered by the individual wanting a specific piece of information. Only if an individual has the metacognitive capacity to “know *what* they don’t know” will their search pattern become indicative of the type of information they are looking for. Therefore, if an individual’s information seeking occurs selectively (when they lack information) and also follows a strategic search pattern (which is suitable to provide the information they lack) then it alludes to the possibility that the individual has the metacognitive awareness that they lack a specific piece of information and is performing a ‘targeted search’ (Call 2012).

Studies into animal’s information seeking abilities typically involve hiding a desired object and measuring how and to what degree the animal will search for the object (Marsh 2019; Subias et al. 2025). In a pioneering study, Call and Carpenter (2001) demonstrated that great apes were selective in their information seeking; which the authors interpreted as evidence that the apes were aware when they did not know the location of a piece of food. The apes had to choose between two tubes, one of which contained a food reward. When the baiting event was hidden, the apes were much more likely to bend down and check the contents of the tubes before choosing. This selectivity in when the apes chose to search, points towards them being sensitive to the fact that they are missing information in order to make a correct choice – in this case the location of the food. Additionally, in a following study by Call (2010), if the tubes were shaken by the experimenter before choosing, then the apes checking reduced, further supporting the idea that they were checking in response to not knowing the location of the food. Using the same paradigm, it has also been shown that when baiting is hidden, macaques will check the contents of tubes before choosing, but when baiting is visible, they will choose instantly (Hampton et al. 2004). However, when similar food-search paradigms have been presented to capuchin monkeys the results have been mixed. Basile et al. (2009) found that when they presented monkeys with 4 opaque tubes, one of which contained food, 4/5 of the monkeys always searched for food irrespective of the baiting condition. Only one capuchin monkey showed a significant tendency to search more often in hidden baiting trials. However, the monkeys in this study underwent a large amount of search training before completing the task and so it is possible they had been inadvertently trained to always search when this apparatus was present. In contrast, Vining and Marsh (2015), gave capuchin monkeys minimal training before having them choose between 2 cups, one of which contained food. The cups were sat on a transparent shelf and so to locate the food during hidden baiting trials the monkeys had to bend down and look up through the shelf. Two of the monkeys looked significantly more often when baiting was hidden.

In all of the studies mentioned above, the object being sought is a food reward. Although this increases the likelihood of the animal being motivated enough to search, it does raise concerns that the strategic food search found is not an example of metacognitive abilities, but rather a reflection of the species foraging strategy. When an animal is hungry it will search for food until it finds it and can satiate its hunger. Therefore, it is possible that in food search tasks the primates are simply following a simple rule of “find the food” meaning that in visible trials no search is required but in hidden trials they search for food without the metacognitive awareness that they don’t know where the food is (Kornell et al. 2007). A study with three language trained chimpanzees attempted to explore this by comparing search patterns when food was vs. wasn’t revealed at the start of trials (Beran et al. 2013). However, the item being sought remained a food reward. This concern around food search was finally addressed in two studies with nonhuman apes (Bohn et al. 2017; Mulcahy 2016). For instance, in the study by Bohn et al. (2017), chimpanzees and orangutans were required to search for tools rather than for food. The ape’s attention was first drawn to an out of reach food item before then being drawn to a choice window. At the choice window the apes could select which tool they wanted to attempt to retrieve the food, however, in some trials the apes were able to see the choices before they were occluded, whereas in other trials they were not - requiring the individuals to peek over a barrier to locate the required tool. The authors found that peeking was more common in trials where the apes didn’t have visual access to the tools beforehand, suggesting that their selective search strategy is not exclusive to food search and therefore may in fact demonstrate a form of metacognition. Methodology such as this is yet to be used with monkeys.

A second concern with current information seeking studies is that for all of the tasks mentioned previously, there is only one possible way to search for information. This adds to the possibility that when the primates do not know where something is they follow a known exploration routine rather than employing a strategic metacognitive information search (Kornell et al. 2007). Conversely, if subjects could be shown to be making rational inferences about where to search, by integrating both known information and knowledge about missing information, this would suggest their information seeking was both flexible and targeted. This would lend plausibility to the idea that it is a deliberate, executive control process, rather than an associatively learned routine (Beran et al. 2016; Beran and Smith 2011; Krachun and Call 2009; Roberts et al. 2009).

In this study we looked for evidence that capuchin monkeys would try to fill gaps in their knowledge by selectively seeking information about food and functionality. We made two significant modifications to the previously used procedures in the hope that it would provide stronger evidence that any information seeking observed was not simply use of a natural foraging strategy. Firstly, we modified the testing setup to give participants multiple ways to seek information. This enabled us to assess whether the monkeys were monitoring their knowledge state and then being strategic in their search. Secondly, if selective information seeking is supported by domain general abilities, then primates should be able to apply their search selectivity to more than just food. Seeking of functional information is yet to be seen in monkeys. To account for the fact that the monkeys in our study population are not as prolific at tool use as chimpanzees or orangutans, our task differed from Bohn et al. (2017) in the way the non-food information was presented. In previous work (Jordan et al. 2021) capuchin monkeys were shown to understand the causal relation between cups and lids, successfully choosing an open cup over a cup with a lid in order to obtain food. We used their success in that task and incorporate it into this study. As well as searching for knowledge about the location of food, monkeys were required to search for information about the functionality of the cups containing the food i.e. whether the cup was sealed or open.

We predict that if monkeys are selective about filling gaps in their knowledge, then they should only seek information when it is missing. Further, if monkeys are strategic about filling gaps in their knowledge, then their seeking pattern should be different in trials which provide differing knowledge gaps. Finally, if monkeys’ information seeking is underpinned by a flexible, domain general metacognitive ability, then they should exhibit similar seeking patterns for both food and functional information.

## Experiment 1: food search

Evidence for selective information seeking in capuchin monkeys is mixed. Only after training did the majority of capuchins show selectivity in Basile et al.’s task (2009), and although all three of the capuchins showed the expected pattern of looking in the basic task in Vining and Marsh’s task (2015), their performances on the following versions were very variable. In both of these studies, the monkeys only had one way to search for information and training was required in order for them to perform the required seeking action. In this study, we use a modification of Vining and Marsh’ (2015) setup that provides the monkeys with multiple ways to search based on behaviour previously seen occurring naturally in the testing room.

### Method

#### Subjects and housing

The capuchin monkeys that participated in the study were housed at the University of St Andrew’s “Living Links to Human Evolution Research” Centre located within the Royal Zoological Society of Scotland’s Edinburgh Zoo. At the Centre the monkeys live in two mixed species communities, with each group made up of common squirrel monkeys (*Saimiri sciureus*) and brown tufted capuchin monkeys (*Sapajus sp.*). Both group’s enclosures consist of an indoor capuchin area (7 m by 4.5 m by 6 m high) to which both species have access, an indoor squirrel monkey enclosure (5.5 m by 4.5 m by 6 m high) to which only the squirrel monkeys have access, and a large shared outdoor area (approximately 900m^2^) consisting of natural vegetation and climbing structures. Situated between the indoor areas is a research room, where, at specified research times the monkeys have access to their testing cubicles. Research sessions took place up to 5 days a week, twice a day at 11.15am – 12.45pm and 2.15pm −4pm Monday-Fridays. Subjects came from both of the groups at the centre; the East group and the West group. The two groups live in adjacent enclosures which are a mirror image of each other, under identical housing conditions and with social groups of similar size. The monkeys are fed a variety of fruits, vegetables, cereals and insects several times per day. The monkeys are never food deprived and water is available ad libitum. Participation in the experiments was voluntary, the monkeys could choose to leave at any time, and all food rewards provided (peanuts, raisins, and dates) were supplemental to the monkeys’ daily diet.

#### Ethics

All research and husbandry complied with the European Association of Zoos and Aquaria (EAZA) and the World Association of Zoos and Aquariums (WAZA) regulations. The research was approved by the School of Psychology and Neuroscience ethics committee at the University of St Andrews as well as the BRU committee at RZSS Edinburgh zoo, consisting of the Zoo Research Liaison Officer, the Scientific Director, and the Research Coordinator.

#### Setup

**Fig. 1:**
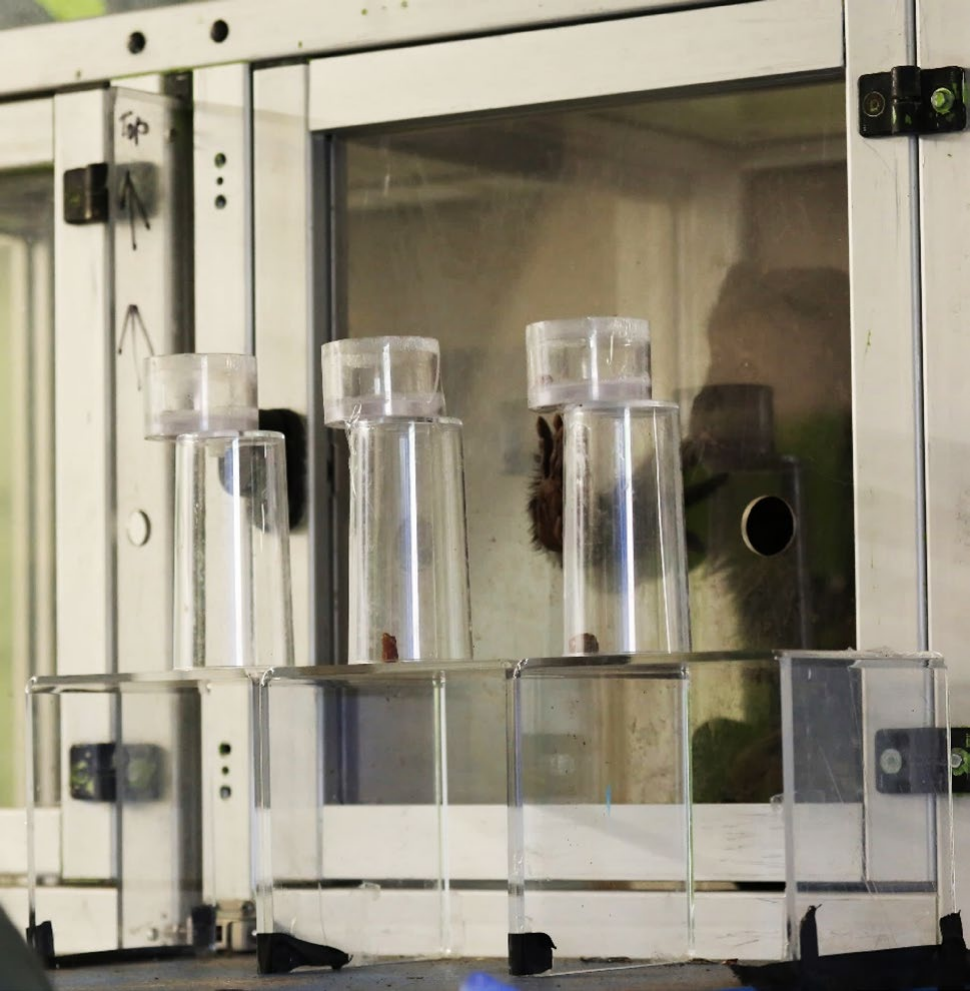
A picture of a set of transparent cups set up for testing

All monkeys were tested in the familiar testing cubicles. Participants entered the testing cubicles and were given access to two adjacent cubicles. In the front side of one of the cubicles (facing toward the experimenter), we installed a Plexiglas panel with 3 evenly spaced holes 10 cm from the bottom. Behind these holes (outside the cubicle) was a transparent plastic stand (15 cm × 15 cm × 45 cm) onto which cups were placed during trials (Fig. 1). The cups comprised of two compartments. Food could be hidden underneath the cup inside the bottom compartment, and/or dropped inside the top compartment. The two compartments were attached slightly unaligned so that the monkeys could not mistake that food dropped in the top would fall through to the bottom. The stand was positioned on a table just out of arms reach of the monkeys, as this was the distance at which, during opaque trials, in order to peek into the top section of the cup, the monkeys had to stand up. The type of food rewards placed in the different compartments of the cups was kept consistent throughout the task for each individual. This meant that for half of the monkeys, the food placed into the top compartment was always a peanut, and the food hidden underneath the cups was always a quarter of a date, with the opposite being true for the other half of the monkeys. In transparent trials a set of transparent cups was used, and in opaque trials an identical, but opaque set was used. In every trial one of the cups only contained food in the top compartment, one only contained food in the bottom compartment, and one contained food in both compartments.

#### Design and procedure

At the beginning of each trial an occluder was placed across the window of the testing cubicle, blocking the monkey’s view of the stand. One by one the cups were then placed on the stand and any hidden baiting occurred. Each cup was placed equidistantly from one another (10 cm apart) in line with the holes in the Plexiglas. The occluder was then removed to reveal the cups on the stand, and any visible baiting was carried out. Once all of the cups were baited the experimenter looked down so as not to cue the monkeys, and a 5 s ‘peeking interval’ began. During the peeking interval the monkeys were able to peek both by standing up and looking down into the top compartment of the cups from above (enabling them to determine which cups contained food in the top) as well as by crouching down and looking up into the cups from below through the transparent stand (enabling them to determine which of the cups contained food in the bottom). After the 5 s had passed, the experimenter faced the participant and said their name followed by “choosing” to signal to the monkey that they should point to their desired cup. All of the monkeys had a large amount of previous experience with choosing cups by pointing through the respective hole. Whichever cup the monkey chose the experimenter lifted to reveal any food in the bottom compartment, and then tipped upside down to empty out any food in the top compartment. Any revealed food was then given to the monkey. If the monkey chose correctly by pointing to the double-baited cup this meant it received both a piece of date and a peanut. Choosing either of the other two cups resulted in the monkey receiving just one of the types of food. After passing the monkey the food it had won, the contents of the other two cups was revealed and moved to a discard pile, out of view of the monkeys.

In order to look for any strategic searching we presented the monkeys with 4 different baiting conditions: All-visible, None-visible, Bottom-only, and Top-only. In All-visible trials, all the baiting occurred after the occluder had been removed so that it was in full view of the monkeys. In None-visible trials, all of the baiting occurred behind the occluder so that removing the occluder started the 5 s peeking interval. In Bottom-only trials, the top compartment of the cups were baited behind the occluder, and the bottom compartment was baited once the occluder was removed. Finally, for Top-only trials the bottom compartment was baited behind the occluder and the top compartment baited once the occluder was removed.

All participants received up to 8 sessions with transparent cups followed by one session with opaque cups. Only monkeys who passed the transparent stage were moved onto the opaque stage. To pass the transparent stage, monkeys had to select the correct cup a minimum of 11/16 times over 2 consecutive sessions (significantly above chance according to binomial with chance levels of 0.33 and alpha set to 0.05). The monkeys received a maximum of 2 sessions per day, 5 days per week, with 16 trials per session. The 16 trials comprised four 4-trial blocks with every 4-trial block including all baiting conditions in a pre-determined random order.

#### Scoring

For cup choice, the trials were live coded by the experimenter. Trials were coded as correct (1) if the monkey chose the target (double-baited) cup and incorrect (0) if any other cup was chosen. For each trial, the peeking was then coded from the video data. We coded the presence/absence of an above peek, and/or a below peek. Above peeking was classed as present if, during the peeking interval, the monkey stood up so that its eyes went above a predetermined critical height and looked towards the cups. The critical height was the height at which the monkey was able to see completely into the top compartment. This was determined before the start of data collection by placing a camera inside the cubicle to get a ‘monkey’s eye view’ of the cups on the outside. Below peeking was classed as present if, during the peeking interval, the monkey crouched down so that its eyes could be seen below the cups, looking up towards their contents. Both types of peeking were recorded as present or absent and these scores used to calculate the binary score for “any peek” – at least one type of peeking present, and “both peek” – both types of peeking present or absent.

A second coder scored 20% of all trials from the recorded video material to establish inter-observer reliability. Fleiss’ kappa was calculated for cup choice, as well as for above and below peeks. According to Landis and Koch (1977), for cup choice and above peeks, inter-observer reliability was “almost perfect” (cup choice: K = 0.96, *p* < 0.001; above peeks: K = 0.85, *p* < 0.001), whist for below peeks, inter-observer reliability was “substantial” (below peeks: K = 0.71, *p* < 0.001).

#### Analysis

All analysis was conducting using R version 4.0.5 (R Core Team 2021). All generalised linear mixed models (GLMM; Baayen 2008) were run using the R packages lme4 (v1.1-26; Bates et al. 2016). Prior to each model, any continuous variables were z-transformed (to a mean of zero and a standard deviation of one) to make the estimates easier to interpret. All models contained trial number within session as a control predictor as well as the random effect of subject ID and the random slopes of the test predictors and trial number (Barr et al. 2013). We tested the effect of the test predictors using likelihood ratio tests comparing the full model with reduced models lacking the respective test predictors. Following any significant results from the GLMMs we ran multiple comparison post hoc tests using the R package multcomp (v.1.4–16; Hothorn et al. 2008).

#### Search behaviour

To analyse the search behaviour of the monkeys, we used the presence of peeking as our DV and ran four separate GLMMs. GLMM1.1 investigated the effect of visibility on the presence of peeking. We predicted that peeking would increase when the information was hidden and so used ‘any peek’ as the DV and visibility condition (transparent or opaque) as the test predictor. GLMMs 1.2a, b, & c, investigated the effect of baiting configuration on the presence of different types of peeking. With these models we wanted to test whether different configurations of missing information would lead to differences in the monkeys’ search patterns. These models used only the data from the opaque trials and contained the test predictor baiting configuration (all visible, top-visible, bottom-visible). For GLMM1.2a, the DV was set to any peek above the barrier, for GLMM1.2b the DV was any peek below the barrier, and for GLMM1.2c the DV was the presence of a double peek (peeking both above and below the barrier). All models used a binomial error structure and logit link function with the variance inflation factors confirming there was no problem of collinearity (for all models VIF = 1 for all fixed effects; Field et al. (2012)).

#### Choice behaviour

To analyse the monkeys’ cup choice, we first ran one-sample t-tests to look at the average scores in each visibility condition compared to chance. Following this we ran a binomial GLMM (GLMM1.3) to investigate the effect of peeking on the monkeys’ cup choice. We predicted that choosing the correct cup would be associated with trials where the monkey’s peeking was sufficient to reveal the information that was missing. For this model we subset the data to remove the transparent and ‘all baiting visible’ trials as in these trials no peeking was necessary to locate the correct cup. We also removed any trials where no peeking had occurred. GLMM1.3 contained cup choice (correct = 1, incorrect = 0) as the DV and the test predictor variables baiting configuration and the presence of an appropriate peek. An appropriate peek was defined as a peek above in the ‘bottom visible’ trials, a below peek in the ‘top visible’ trials and a double peek (both above and below) in the ‘none visible’ trials. An appropriate peek was recorded regardless of the presence of extra superfluous peeks. Both models used a binomial error structure and logit link function with the variance inflation factors confirming there was no problem of collinearity (for both models VIF = 1 for all fixed effects; Field et al. 2012).

### Results

All nine monkeys who chose to participate in Experiment 1 passed the visible stage and moved onto the opaque stage.

#### Search behaviour

**Fig. 2:**
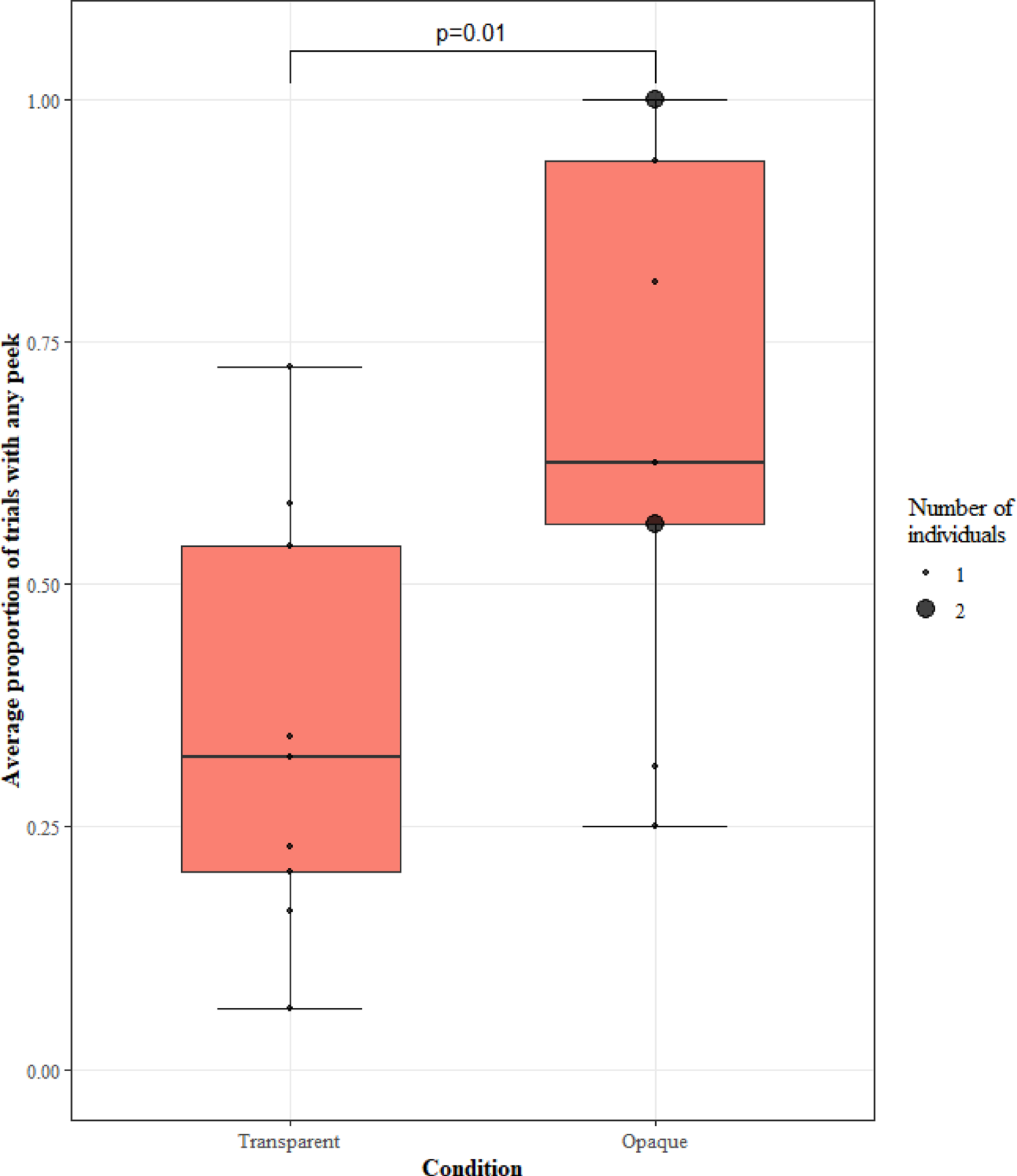
The proportion of trials with any peeks (including top and below peeks) for each visibility condition. Black circles show the average for individual monkeys, with the size of the point representing the number of monkeys

We looked at any effect of visibility on the peeking frequency of the monkeys to test the prediction that the occluded condition would be associated with higher levels of peeking. The model (GLMM1.1 with any peeking as the DV) was significant when compared to a null model lacking the test predictor (LRT: χ2 = 6.51, df = 1, *p* = 0.01; Fig. 2) with peeking frequency significantly increasing in the opaque sessions compared to the transparent sessions.

**Fig. 3:**
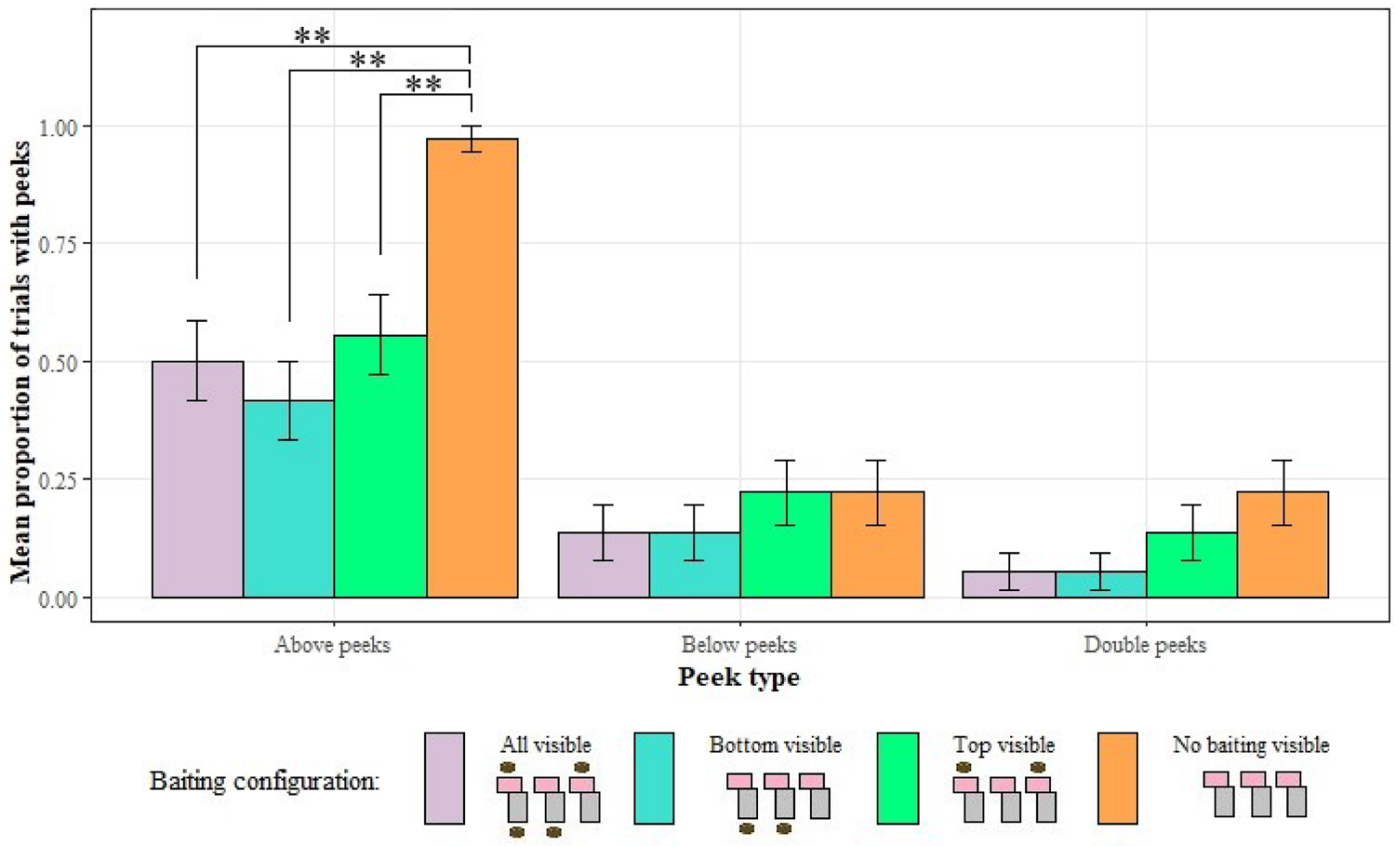
The proportion of trials with each type of peek for each of the baiting configurations in opaque sessions. Error bars show the mean ± se. Tukey HSD: **p < 0.01

Next, to test whether different configurations of missing information would lead to differences in the monkeys’ search patterns we looked for any effect of baiting configuration on the frequency of peeking during the opaque trials. We used the data from opaque trials only and ran three separate binomial GLMMs with baiting configuration as the test predictor. The models with the presence of below and double peeks as the DV (GLMM1.2b and 1.2c) were not significantly different to their respective null models lacking the test predictor (*p* > 0.05). However, the model with above peeks as the DV (GLMM1.2a) was significant when compared to a null model lacking the test predictor (LRT: χ2 = 15.40, df = 3, *p* = 0.002), suggesting an effect of baiting configuration on above-peek frequency. Post hoc analysis found no significant differences between baiting conditions where at least one form of baiting was visible, but some indication that the effect was caused by the ‘no visible baiting’ condition. (Tukey HSD: all–bottom: z = 0.99, *p* = 0.74; all–top: z= −0.71, *p* = 0.88; bottom–top: z = −1.63, *p* = 0.34; all–none: z = −3.97, *p* < 0.001; bottom–none: z = −4.29, *p* < 0.001; top–none: z = −3.71, *p* < 0.001; Fig. 3).

#### Choice behaviour

In both visibility conditions the monkeys chose the correct cup (double-baited) above chance (one-sample t-tests: transparent: t(8) = 14.31, *p* < 0.001; opaque: t(8) = 4.92. *p* = 0.001). Although the monkeys scored above chance in both conditions, performance in the transparent condition (*M* = 0.61, *SE* = 0.02) was above that in the opaque condition (*M* = 0.5, *SE* = 0.04).

Next, we looked at whether monkeys were using their peeking to help them make a correct choice during the opaque trials. The model (GLMM1.3) was not significant when compared to a null model lacking the test predictors (LRT: χ2 = 4.04, df = 3, *p* = 0.26), suggesting no effect of appropriate peeking on making a correct choice. Performance in trials with an appropriate peek (*M* = 0.58, *SE* = 0.09) was not significantly different to performance in trials without an appropriate peek (*M* = 0.48, *SE* = 0.06).

### Discussion

Overall, we found evidence for selectivity in the monkeys’ information seeking as the monkeys exhibited more of the peeking behaviour when there was information missing. However, looking at the patterns of their peeking in more detail did not reveal any evidence that their information seeking was performed strategically. As a group, the monkeys were able to choose the correct cup above chance in both the transparent and opaque conditions, however in the opaque condition those who performed appropriate peeking weren’t any better at choosing the correct cup than those who did not.

The monkeys were much more likely to perform an above peek, and during data collection we noticed that monkeys began to peek as soon as the occluder was removed irrespective to whether baiting was complete. This meant that during the peeking interval we could not be sure what information they already had about the contents of the cups. For some monkeys their peeking during the baiting meant that they missed the baiting information, whilst for others they gathered all the information they needed during the baiting. In Experiment 2 we attempted to correct this by running a follow up session.

## Experiment 2: food search follow up

In Experiment 1, the monkeys started peeking as soon as the occluder was removed. This meant we were unable to ascertain what information they had gathered about the baiting. In Experiment 2 we adjusted our set up to prevent the monkeys from gathering information until all the baiting was complete.

### Method

#### Subjects and housing

The same nine monkeys who participated in Experiment 1 underwent one additional session for Experiment 2.

#### Setup and procedure

The setup and procedure were identical to the Opaque session of Experiment 1 with one exception. The table which we stood the cups on was pulled back away from the testing window to a distance where any attempt from the monkeys to stand up or crouch down to look into the cups did not lead to them being able to see the contents of the cups. The table remained at this distance until all hidden and visible baiting was complete, before being push forwards (back to its original position) in order to start the 5 s peeking interval.

#### Scoring

For the cup choice, the trials were live coded by the experimenter. As in Experiment 1, trials were coded as correct (1) if the monkey chose the target (double-baited) cup and incorrect (0) if any other cup was chosen. For each trial, the peeking was then coded from the video data. The same coding scheme as Experiment 1 was used with the peeking interval beginning once the table was moved into place and ended once the experimented asked the monkey to choose a cup.

A second coder scored 33% of all trials from the recorded video material to establish inter-observer reliability. Fleiss’ kappa was calculated for cup choice, as well as for above and below peeks. According to Landis and Koch (1977), for cup choice inter-observer reliability was “almost perfect” (cup choice: K = 1 *p* < 0.001), whist for above and below peeks, inter-observer reliability was “substantial” (above peeks: K = 0.78, *p* < 0.001; below peeks: K = 0.76, *p* < 0.001).

#### Analysis

Analysis followed the same structure as in Experiment 1.

#### Search behaviour

As in Experiment 1, to investigate whether different configurations of missing information would lead to differences in the monkeys’ search patterns, GLMMs2.1a, b, & c all included the test predictor variable baiting condition. For GLMM2.1a we used any peek above the barrier as the DV, for GLMM2.1b we used any peek below the barrier as the DV, and for GLMM2.1c we used the presence of a double peek (peeking both above and below the barrier) as the DV.

#### Choice behaviour

To analyse the monkey’s choice performance, we ran one-sample t-tests to compare their performance to chance. Following this GLMM2.2 used cup choice (correct = 1, incorrect = 0) as the DV, and included the test predictor variables baiting configuration and the presence of an appropriate peek to investigate whether monkeys were using their peeking to help them make a correct choice during the opaque trials.

GLMMs 2.1–2.2 all used a binomial error structure and logit link function and contained trial number within session as a control predictor as well as the random effect of subject ID and the random slopes of the test predictors and trial number within the random effect of subject ID. VIF for all models confirmed that there was no collinearity between their predictors as for all predictors VIF = 1.

### Results

#### Search behaviour

**Fig. 4:**
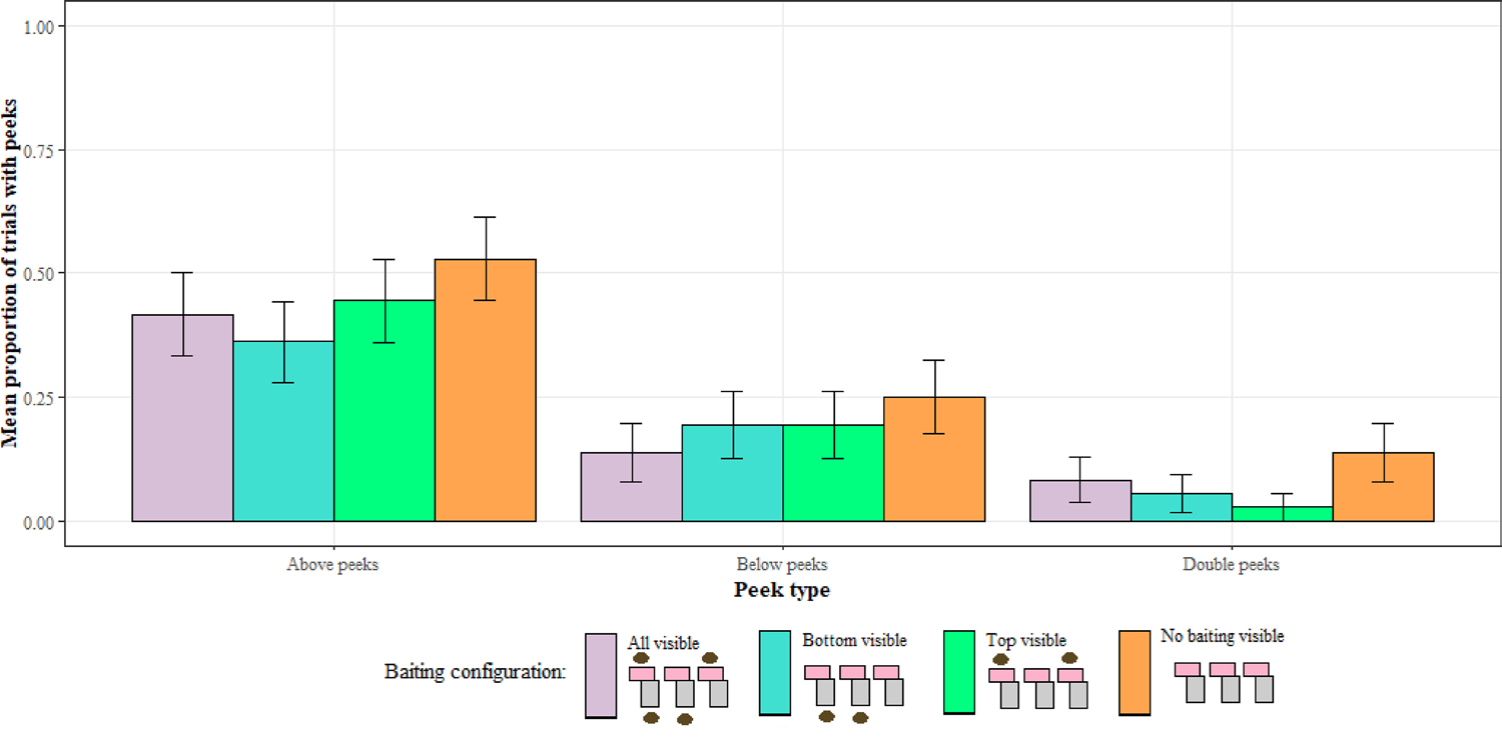
The proportion of trials with each type of peek for each of the baiting configuration in opaque trials in Experiment 2

To look for any effect of baiting configuration on the frequency of peeking and explore whether monkeys’ search patterns reflected the information gap they were presented with, we ran three separate GLMMs. GLMM2.1a had the DV set to any peek above the barrier, GLMM2.1b had the DV set to any peek below the barrier, and GLMM2.1c had the DV set to the presence of a double peek (peeking both above and below the barrier). None of the three models were significantly different to their respective null models (for all models *p* > 0.05; Fig. 4).

#### Choice behaviour

The monkeys continued to choose the correct cup (double-baited) above chance (one-sample t-tests: t(8) = 4.52. *p* = 0.002), with performance similar to that in Experiment 1 (*M* = 0.47, *SE* = 0.04).

**Fig. 5:**
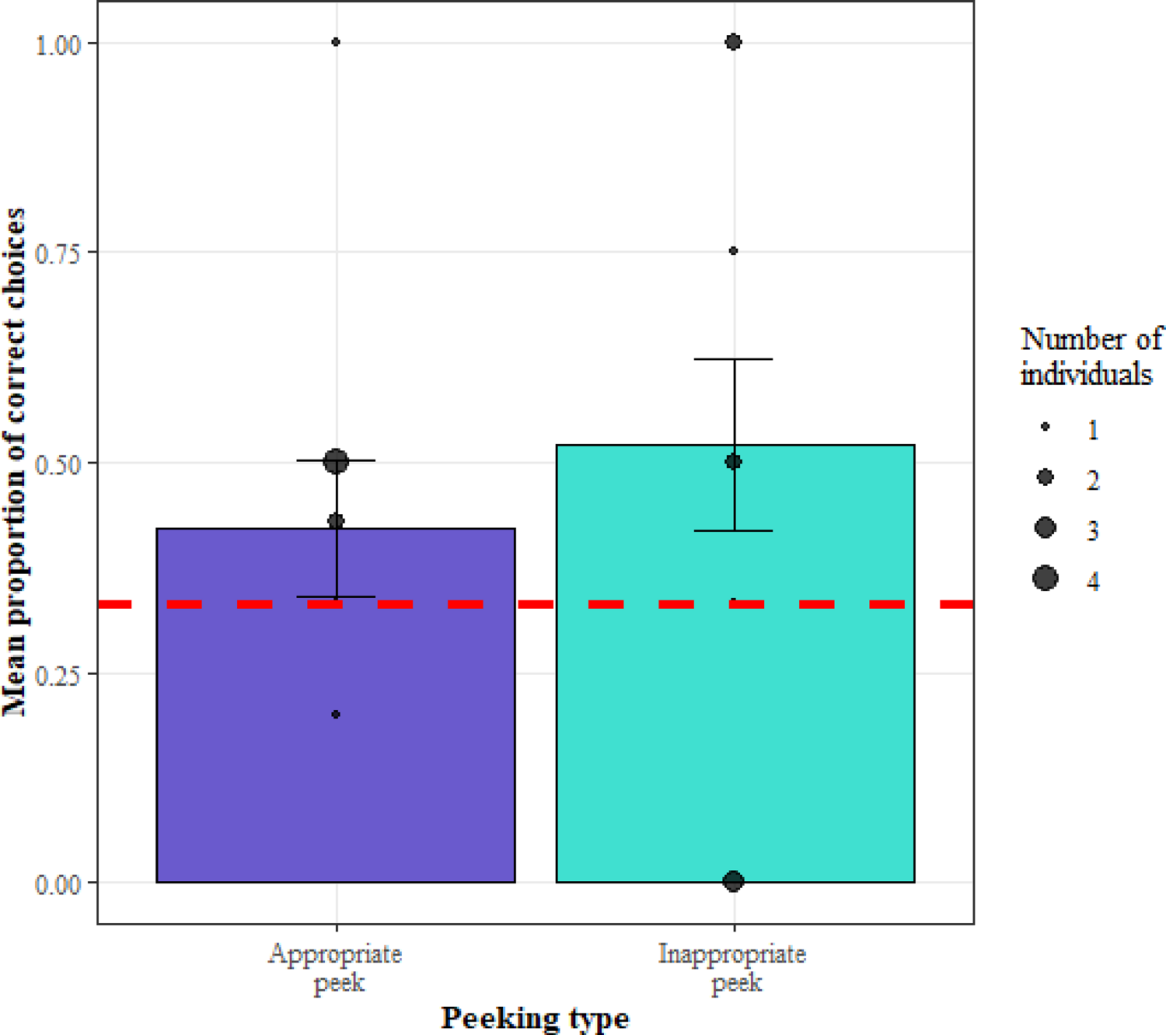
The average proportion of correct choices in trials with inappropriate and appropriate peeking in Experiment 2. The error bars show the mean ± se with the black circles showing average for individual monkeys and the size of the point showing the number of monkeys at any one location. The red dashed line indicates chance

We then investigated whether monkey’s who performed appropriate peeks were more likely to choose the correct cup. The model (GLMM2.2) was not significant when compared to a null model (LRT: χ2 = 4.10, df = 3, *p* = 0.25), suggesting no effect of appropriate peeking on making a correct choice (Fig. 5). Performance in trials with an appropriate peek (*M* = 0.52, *SE* = 0.10) was not significantly different to performance in trials without an appropriate peek (*M* = 0.46, *SE* = 0.06).

### Discussion

Changing the position of the table to prevent the monkey’s from gathering information about the contents of the cups before the peeking interval did not reveal any extra selectivity in their peeking patterns. As a group, the monkeys continued to choose the correct cup above chance with those who performed appropriate peeking not significantly better at choosing the correct cup than those who did not.

## Experiment 3: food and functionality search

When looking for food information in Experiments 1 and 2, monkeys were selective if not strategic. However, to provide evidence that this is not purely a foraging strategy, in Experiment 3 the monkeys were required to search for information pertaining to the functionality of the cup as well as the location of the food. Although some populations of wild capuchin monkeys use tools (Chevalier-Skolnikoff 1990; Fragaszy and Cummins-Sebree 2005; Lima et al. 2024), the capuchin monkeys participating in this study have never been observed using tools. Therefore, we designed a new set up in which the monkeys had to search for non-food information in order to receive a food reward. In previous work (Jordan et al. 2021), the capuchins in this study had taken part in a study on causal understanding where they had to learnt to avoid cups with lids which trapped a food reward. Twelve out of 14 monkeys successfully passed this task within 10 trials, learning to avoid the cup with a lid. For the current study we adapted this task so that before selecting a cup the monkeys would have to search for information about the presence of the lid.

Additionally, in Experiment 1 we found that many of the monkeys were beginning to peek as soon as the occluder was removed irrespective of whether all cups were fully baited. This was problematic for the monkeys as it meant that often they missed out on the information which was supposed to be available during the visible baiting (i.e. the location of some of the food rewards), and led to their peeking often being ineffective at helping them choose the correct cup. We took this into account in Experiment 3 and modified the setup so that in all trials the peeking interval began as soon as the occluder was removed.

Lastly, in Experiments 1 and 2 the risk associated with making a wrong choice was relatively low as the monkeys received a reward of at least one piece of food in every trial. Capuchin monkeys are extractive foragers and have been shown to have a high tolerance for risk (Beran et al. 2016). It has been suggested that this high risk-tolerance has an impact in their performance on cognitive tasks as only in tasks when the risk of failure is high will monkeys employ their more demanding cognitive abilities (Beran et al. 2016; Smith et al.^2018^). Therefore, in Experiment 3 only the correct choice led to the monkeys’ receiving a food reward, and we increase the risk of losing by adding a fourth cup option. For each trial, this decreases the likelihood of winning a food reward from 100% in Experiment 1 down to just 25% in Experiment 3.

For this experiment we predicted that, as in Experiment 1, if monkeys are selective about filling gaps in their knowledge, then they should only seek information when it is missing. Further, if monkeys are strategic about filling gaps in their knowledge, then their seeking pattern should be different in trials which elicit different gaps in their knowledge. Finally, if monkeys’ information seeking is underpinned by a flexible, domain general metacognitive ability, then they should continue to exhibit the selective seeking patterns seen in Experiment 1, despite the search now pertaining to both food and functional information.

### Method

#### Subjects and housing

Sixteen monkeys participated in Experiment 3. Eight of them were the participants from Experiments 1 and 2, whilst eight of them chose to only participate in Experiment 3. The East group received Experiments 1 and 2 followed by Experiment 3, the West group received Experiment 3 followed by Experiments 1 and 2 to control for any priming that could have occurred.

#### Setup

The set up was very similar to Experiment 1, except that the Plexiglas panel in the front side of the cubicle contained 4 evenly spaced holes rather than 3. Additionally, the transparent plastic stand onto which the cups were placed during trials was narrower to allow the use of different cups. During all trials the same large occluder from Experiments 1 & 2 was used to hide the cups from view whilst they were placed onto the stand. In addition, in occluded trials the cups were covered with small individual occluders to block the visual access to the cups once the large occluder was removed. The stand positioning was only slightly different (placed 5 cm away from the window) as this was the distance at which, during occluded trials, the monkeys could stand up and peek over the individual occluders without being able to see the reward in the bottom of the cups.

**Fig. 6:**
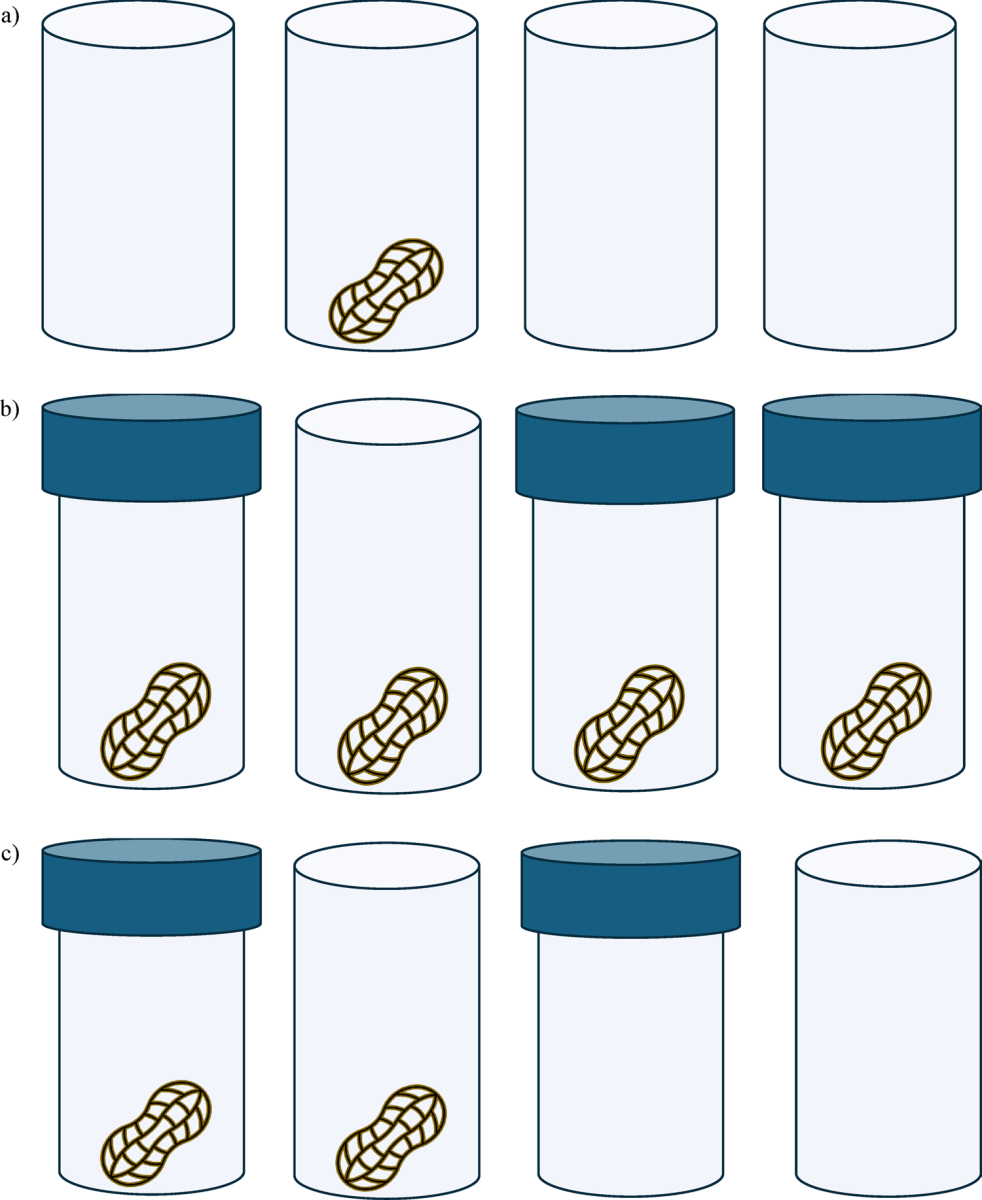
Illustration of the three cup configurations. (**a**) All open configuration: none of the cups have lids but only one cup contains a reward; (**b**) All baited: all four cups contain a reward, but only one cup doesn’t have a lid; (**c**) Mixed: one cup contains a reward but also has a lid, one cup contains a reward and doesn’t have a lid, one cup has a lid but contains no reward, and one cup doesn’t have a lid but doesn’t contain a reward

For Experiment 3, regular transparent cups with just one compartment were used. However, three different cup configurations were presented to the monkeys; All open, All baited, and Mixed (Fig. 6.). In All open trials the monkeys were presented with 4 cups *without* lids, however only one of these cups contained a reward, meaning just one cup was both open and baited. In All baited trials the monkeys were presented with 4 cups all of which contained a reward, however three of the cups were sealed with blue lids, leaving just one cup both open and baited. In Mixed trials the monkeys were presented with 4 cups; one cup without a lid, but also without a reward; one cup with a lid but which didn’t contain a reward; one cup with a lid but which did contain a reward; and one cup without a lid which did contain a reward. This meant that in every cup configuration there was an open-baited cup which was the correct cup to choose to obtain the food reward.

#### Design and procedure

Participants received two conditions: visible and occluded. In the visible condition the occluder was removed to reveal the cups in full view, however in the occluded condition before removing the large occluder, the cups were covered with small individual occluders to block the visual access to the cups once the large occluder was removed. All participants received up to 8 visible sessions followed by just one occluded session. Only monkeys who passed the visible condition were moved onto the occluded condition. In order to pass the visible condition, monkeys had to select the correct cup in 17/24 trials over 2 consecutive sessions (significantly above chance according to binomial with chance at 0.25 and alpha set to 0.05). The monkeys received a maximum of 2 sessions per day, 5 days per week, with 12 trials per session. The 12 trials were made up of 4 blocks of three trials each (one trial of each configuration presented in a pre-determined random order).

At the beginning of a trial, the required cups were placed on a stand in front of the cubicle without the 4-hole Plexiglas window, so that the monkeys could see which cups were available to them for this trial, and also see the open-baited cup being baited. After baiting a large occluder was positioned in front of the adjacent 4-hole window to block the monkey’s view of the second stand. The cups were then moved one by one from the visible stand over to the occluded stand and arranged in a random order. Each cup was placed equidistantly from one another (5 cm apart) in line with the holes in the Plexiglas. Once in place, for occluded trials the cups were covered with small individual occluders to block the visual access to the cups once the large occluder was removed. The large occluder was then removed and, before being allowed to choose a cup, the monkey was given a 5 s ‘peeking interval’ where the experimenter looked away. During the peeking interval the monkeys were able to peek both by standing up and looking down on the cups from above (enabling them to determine which cups did not have lids) as well as by crouching down and looking up on the cups from below through the transparent stand (enabling them to determine which of the cups contained food). After the 5 s had passed, the experimenter faced the participant and said their name followed by “choosing” to signal to the monkey that they should point to their desired cup. All of the monkeys had a large amount of previous experience with choosing cups by pointing through the respective hole. If the monkey chose correctly by pointing to the open-baited cup the experimenter tipped the cup towards the monkey so it could reach in and retrieve the reward. However, if the monkey pointed to one of the incorrect cups, the experiment tipped the cup towards the monkey but either the cup contained no reward, or the blue lid blocked the monkey from retrieving the reward. The cup was then placed back on the holding stand and the experimented picked up the correct cup and tipped the reward into her own hand before placing it into a discard pile, out of view of the monkeys. All of the cups were then placed back onto the holding stand so that the next trial could begin. In each trial only one of the cups was correct.

#### Scoring

The scoring was identical to Experiments 1 and 2. Cup choice was live coded by the experimenter, with trials coded as correct (1) if the monkey chose the target (open-baited) cup and incorrect (0) if any other cup was chosen. Peeking was then coded from the video data, with the criteria for both above and below peeks the same as in Experiment 1. As previously, both types of peeking were recorded absent or present and these scores used to calculate the binary score for “any peek” – at least one type of peeking present, and “both peek”– both types of peeking present.

A second coder scored 20% of all trials from the recorded video material to establish inter-observer reliability. Fleiss’ kappa was calculated for cup choice, as well as for above and below peeks. According to Landis and Koch (1977), for cup choice, inter-observer reliability was “almost perfect” (cup choice: K = 0.99, *p* < 0.001), whist for both above and below peeks, inter-observer reliability was “substantial” (above peeks: K = 0.76, *p* < 0.001; below peeks: K = 0.73, *p* < 0.001).

#### Analysis

Analysis followed the same structure as Experiment 1.

#### Search behaviour

To test whether the monkeys peeked more in response to missing information, we looked for any effect of occlusion on the peeking frequency of the monkeys. As in Experiment 1, we used the presence of peeking as our DV and ran four separate GLMMs. GLMM3.1 investigated the effect of visibility on the presence of peeking with ‘any peek’ as the DV and occlusion condition (visible or occluded) as the test predictors. GLMMs 3.2a, b, & c, investigated the effect of cup configuration on the presence of different types of peeking. These models used only the data from the occluded trials and contained the test predictor cup configuration (all baited, all open, mixed). For GLMM3.2a, the DV was set to any peek above the barrier, for GLMM3.2b the DV was any peek below the barrier, and for GLMM3.2c the DV was the presence of a double peek (peeking both above and below the barrier).

#### Choice behaviour

To analyse the monkeys’ cup choice, we ran one-sample t-tests to look at the average scores in each occlusion condition compared to chance. As in Experiment 1, we then investigated whether monkeys were using their peeking to help them make a correct choice. After removing the visible trials and any trials where no peeking had occurred, GLMM3.3 used cup choice (correct = 1, incorrect = 0) as the DV, and included the test predictor variables cup configuration and the presence of an appropriate peek.

GLMMs 3.1–3.3 all used a binomial error structure and logit link function with the variance inflation factors confirming there was no problem of collinearity (for all models VIF = 1 for all fixed effects; Field et al. 2012).

### Results

All sixteen monkeys who chose to participate passed the visible trials and moved onto the occluded trials.

#### Search behaviour

**Fig. 7:**
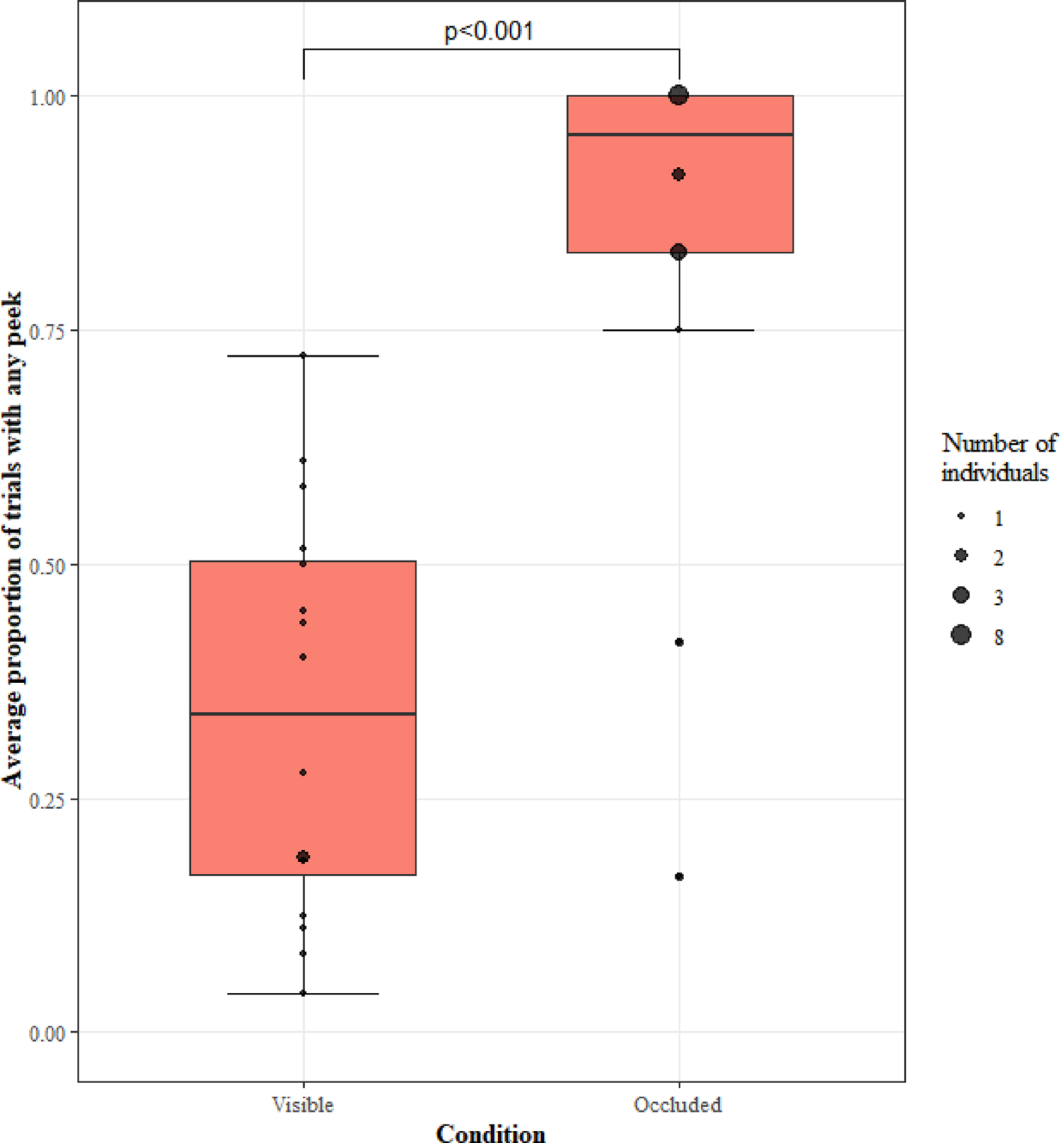
The proportion of trials with peeks for each visibility condition. Black circles show the average for individual monkeys, with the size of the point representing the number of monkeys

We looked at any effect of occlusion on the peeking frequency of the monkeys. The model (GLMM3.1) was significant when compared to a null model, with peeking frequency significantly increasing in the occluded sessions compared to the visible sessions (LRT: χ2 = 23.08, df = 1, *p* < 0.001; Fig. 7).

**Fig. 8:**
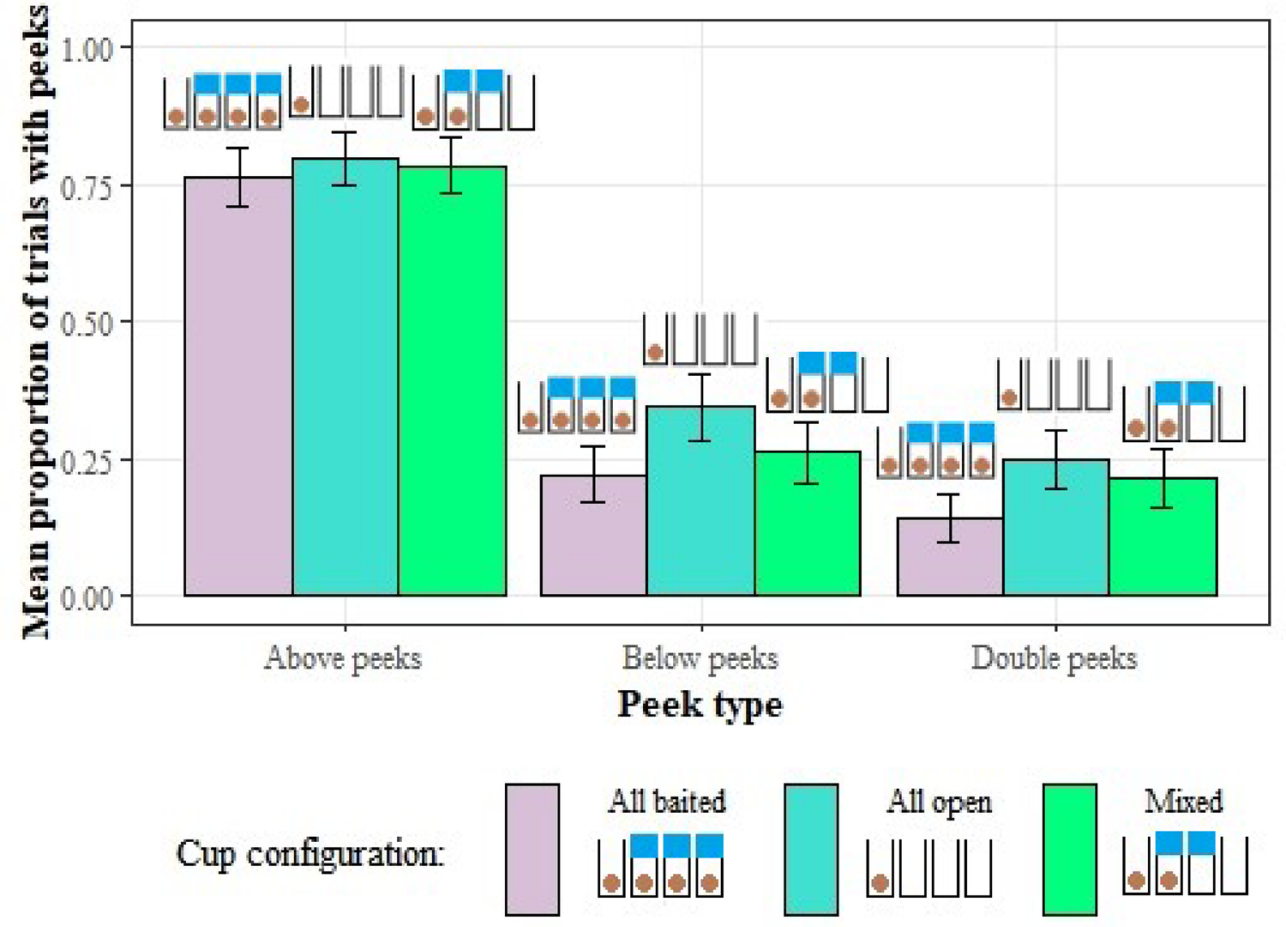
The proportion of trials with each type of peek for each of the baiting configurations. Error bars show the mean ± se

Next, to investigate if the monkeys peeking reflected the missing information, we looked for any effect of cup configuration on the frequency of peeking during the occluded trials. None of the three models (GLMM3.2a, b, & c) were significantly different to their respective null models (*p* > 0.05; Fig. 8.).

#### Choice behaviour

In both conditions the monkeys chose the correct cup (open-baited) above chance (visible: t(15) = 28.35, *p* < 0.001; occluded: t(15) = 4.61. *p* < 0.001). Although the monkeys scored above chance in both conditions, performance in the transparent condition (*M* = 0.68, *SE* = 0.02) was clearly above that in the opaque condition (*M* = 0.36, *SE* = 0.03).

**Fig. 9:**
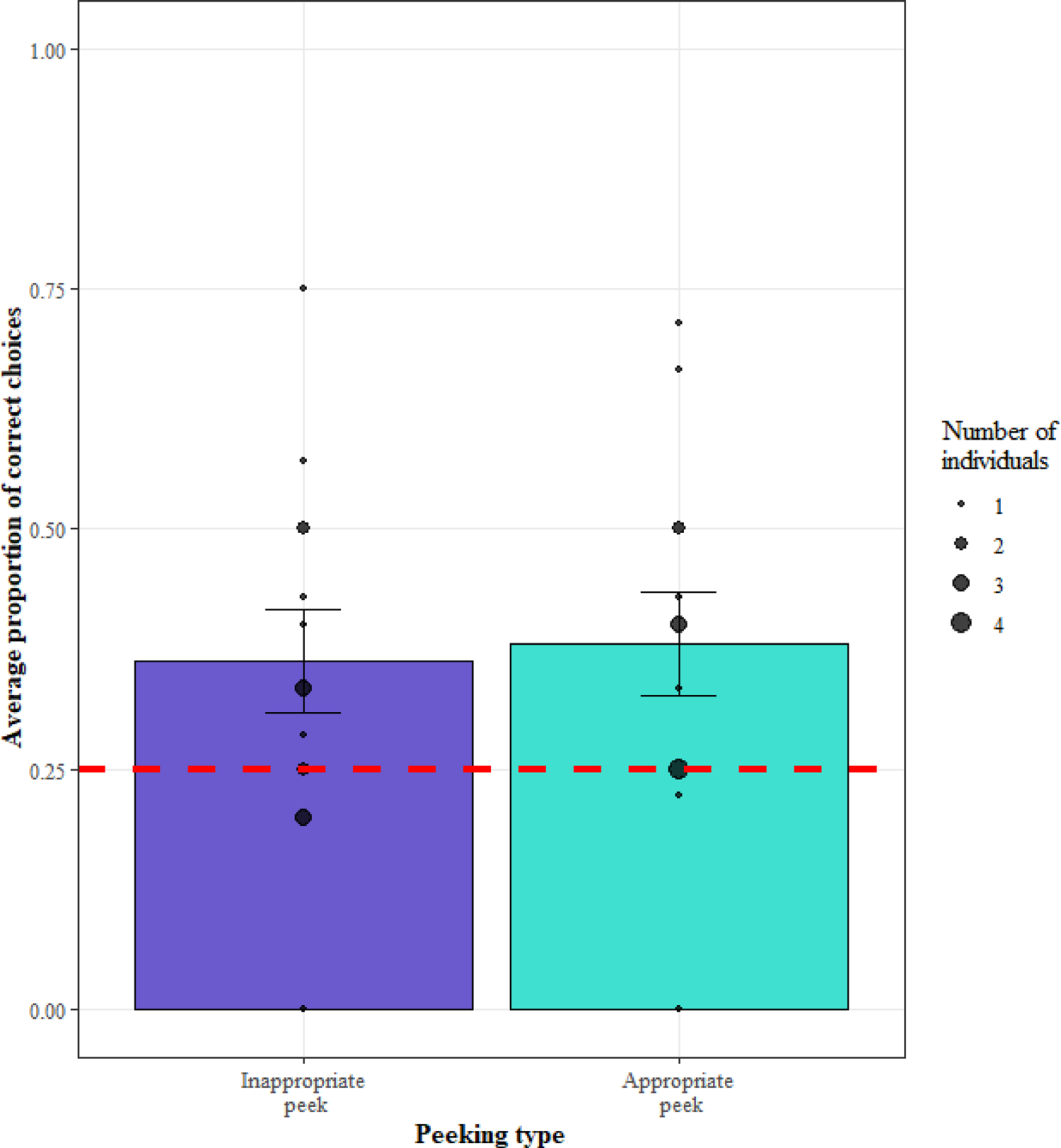
The average proportion of correct choices in trials with inappropriate and appropriate peeking. The error bars show the mean ± se with the black circles showing average for individual monkeys and the size of the point showing the number of monkeys at any one location. The red dashed line indicates chance

We then looked at whether monkeys who performed the appropriate peeks were better at choosing the correct cup. The model (GLMM3.3) was not significant when compared to a null model (LRT: χ2 = 3.46, df = 4, *p* = 0.48), suggesting no effect of appropriate peeking on making a correct choice (Fig. 9.). Performance in trials with an appropriate peek (*M* = 0.38, *SE* = 0.05) was no different to performance in trials without an appropriate peek (*M* = 0.34, *SE* = 0.05).

### Discussion

The monkeys chose the correct cup (open-baited) above chance in both conditions. Their peeking showed selectivity as the rates of peeking were significantly higher in the opaque condition. These results provide evidence that capuchin monkey’s ability to seek information selectively is a flexible, domain general ability as they selectively sought information about the function of the cups as well as about the location of food. However, as cup configuration had no effect on the monkey’s pattern of peeking, in line with our finding in Experiments 1 and 2, we found no evidence that the monkeys fill gaps in their knowledge strategically.

## General discussion

In all three experiments the monkeys showed selectivity in their information seeking, i.e., when information was missing, they showed significantly higher search rates. This selectivity was present both when they were seeking information about food and about the functionality of the cups which suggests their information seeking is a flexible domain general ability and not simply a foraging strategy. Despite showing selectivity in when they sought information, throughout the study the monkeys failed to show any strategic searching as in neither Experiments 1, 2, or 3 did we find any evidence that they were tailoring their search pattern to the missing information. This suggests that although the monkeys may be aware or feel uncertain when they lack information, they may be unaware of the details of the missing information and/or how to fill this gap in their knowledge.

The finding that monkeys peeked more in the opaque and occluded sessions indicates that when there was information missing, the monkeys acted to seek it out. This selectivity in their information seeking could suggest that they are aware when they lack information and therefore, that they possess some form of metacognitive abilities. This is in line with previous work by Vinning and Marsh (2015) which showed that when the baiting event was hidden capuchin monkeys were significantly more likely to attempt to look into the cups before making their choice between the two. However, this increase in search behaviour does not necessarily mean that they have a representation of their knowledge which they then metacognitively reflect on (Proust 2019). Instead, when the monkeys were unable to see the correct choice in the opaque and occluded trials, they may have experienced ‘metacognitive feelings’ that led to metacognitive control, rather than them having an ability for conceptual metacognition (Proust 2019). Being unable to see which cup to choose could have increased their feeling of uncertainty, which led them to perform additional information seeking to lower this uncertainty. This is in line with a study on macaques which showed that they monitor their uncertainty and will show voluntary checking behaviour in response to anxiety inducing uncertainty levels (Bosc et al. 2017).

This distinction is important because the tendency to search more when feeling uncertain can be explained without the need to reflect on one’s own knowledge states.

In all three of our experiments, the monkeys showed high rates of peeking and in addition, the finding that this occurs for both food (Experiments 1 and 2) and for functional information (Experiment 3), suggests that this selectivity is a flexible and domain general response to missing information. This finding is, to our knowledge, the first example of monkeys selectively searching for functional information, and thus rules out the counterargument that their searching behaviour is purely a foraging strategy (Kornell et al. 2007). In the all-baited trials of Experiment 3, monkeys did not need to peek in order to locate the food rewards as all locations contained a reward. However, the monkeys continued to perform the peeking behaviour. This could suggest that when the information gap concerned not the location of food, but the functionality of the cup, they were just as motivated to search for information to fill this knowledge gap and locate the functional (open) cup. Furthermore, in Experiment 3, only a peek below was able to provide food information to the monkeys as peeking above the occluder only revealed the presence/absence of lids on each of the cups, yet the number of trials containing a below peek appeared to be lower than that containing an above peek (Fig. 7). If the monkeys were performing peeks due to following a simple rule of “find the food” (as suggested by Kornell et al. 2007), then we would expect the number of below peeks to be significantly higher as even after performing an above peek they would not have located the food and so should have continued to search.

Despite the propensity for peeking remaining high in the opaque trials of Experiments 1 and 2 and the occluded trials of Experiment 3, the different baiting and cup configurations did not affect the monkeys peeking locations in a meaningful way. We found no patterns in their peeking which suggested they were peeking in order to fill a specific identified gap in their knowledge. The style of information seeking seen in the capuchins in this paper pairs well with claims that surprise functions as a metacognitive signal for a lack of knowledge (Munnich and Ranney 2019) and that in capuchin monkeys their metacognitive abilities act to show the strength of a memory trace rather than a representation of specific details of the memory (Fujita 2009; Beran and Smith 2011; Takagi and Fujita 2018). The memory flag hypothesis (Hampton 2005) suggests that monkeys could have an awareness of their own mental states, but only at the level of memory strength. This means that the capuchins could be aware of the presence of information in their memory without being aware of the details of the memory. The results found by Fujita (2009) supported this hypothesis in capuchin monkeys as when presented with a delayed match to sample test, the monkeys were more likely to skip tests when they hadn’t seen the sample picture before, and when the delay was longer. In line with this finding, the results obtained in this study suggest that although the monkeys were aware of their own knowledge gaps (when they lacked information), they were not aware of the specific information they required to fill these gaps.

In conclusion, the capuchins did exhibit information seeking in response to a gap in knowledge. Their information seeking pertained to both food and functional information; however, their search patterns did not reveal any sensitivity to the specific information that was missing. Therefore, we conclude that the capuchin monkeys can detect a gap in their knowledge, either through an increased feeling of uncertainty and/or an awareness of lack of knowledge and have the flexibility in their information seeking to explore in response to this. However, it is likely that their metacognitive abilities enable them to be aware of a lack knowledge or increased feelings of uncertainty, without being aware of the contents of their memory trace or what information is required to fill the gap.

## Supplementary Information

Below is the link to the electronic supplementary material.

Supplementary Material 1

## Data Availability

The data generated and analysed during this study are published on Figshare (https://figshare.com/projects/Capuchin_peeking/243848), alongside the source code for the analysis.
